# Metabolic Resistance in Permethrin-Resistant Florida *Aedes aegypti* (Diptera: Culicidae)

**DOI:** 10.3390/insects12100866

**Published:** 2021-09-24

**Authors:** Sierra M. Schluep, Eva A. Buckner

**Affiliations:** Florida Medical Entomology Laboratory, Department of Entomology and Nematology, Institute of Food and Agricultural Sciences, University of Florida, Vero Beach, FL 32962, USA; sschluep@ufl.edu

**Keywords:** permethrin, pyrethroids, *Aedes aegypti*, metabolic resistance, inhibitors, PBO

## Abstract

**Simple Summary:**

Pyrethroid-oriented vector control programs have increased worldwide to control adult *Aedes aegypti* mosquitoes and quell *Aedes*-borne disease epidemics. Due to years of pyrethroid use, resistance to pyrethroids in *Ae. aegypti* has become a global issue. In Florida, permethrin is the most common pyrethroid adulticide active ingredient used to control mosquito populations. Thus far, all wild Florida *Ae. aegypti* populations tested against permethrin have been found to be resistant. Metabolic resistance is a major mechanism of resistance in insects in which enzyme-mediated reactions cause the degradation or sequestration of insecticides. We performed assays to investigate the presence of metabolic resistance in 20 Florida *Ae. aegypti* populations and found that 11 populations (55%) exhibited metabolic resistance due to the action of at least one of three classes of metabolizing enzymes: oxidases, esterases, and glutathione transferases. Additionally, we identified two metabolic enzyme inhibitors: S.S.S-tributyl phosphorotrithioate (DEF; inhibits esterase activity) and diethyl maleate (DM; inhibits glutathione transferase activity), in addition to the commonly used piperonyl butoxide (PBO; inhibits oxidase activity), which were able to increase the efficacy of permethrin against resistant *Ae. aegypti* populations. Pre-exposure to DEF, PBO, and DM resulted in increased mortality after permethrin exposure in eight (73%), seven (64%), and six (55%) of the *Ae. aegypti* populations, respectively. Increasing the effectiveness of pyrethroids is important for mosquito control, as it is the primary method used for adult control during mosquito-borne disease outbreaks. Considering that DEF and DM performed similarly to PBO, they may be good candidates for inclusion in formulated pyrethroid products to increase their efficacy against resistant mosquitoes.

**Abstract:**

*Aedes aegypti* is the principal mosquito vector for many arthropod-borne viruses (arboviruses) including dengue, chikungunya, and Zika. In the United States, excessive permethrin use has led to a high frequency of resistance in mosquitoes. Insecticide resistance is a significant obstacle in the struggle against vector-borne diseases. To help overcome metabolic resistance, synergists that inhibit specific metabolic enzymes can be added to formulated pyrethroid products. Using modified CDC bottle bioassays, we assessed the effect of three inhibitors (piperonyl butoxide (PBO), which inhibits oxidase activity; S.S.S-tributyl phosphorotrithioate (DEF), which inhibits esterase activity; and diethyl maleate (DM), which inhibits glutathione transferase activity) + permethrin. We performed these against 20 Florida *Ae. aegypti* populations, all of which were resistant to permethrin. Our data indicated that 11 out of 20 populations (55%) exhibited metabolic resistance. Results revealed 73% of these populations had significant increases in mortality attributed to DEF + permethrin, 64% to PBO + permethrin, and 55% to DM + permethrin compared to permethrin alone. Currently, PBO is the only metabolic enzyme inhibitor added to formulated pyrethroid products used for adult mosquito control. Our results suggest that the DEF and DM inhibitors could also be useful additives in permethrin products, especially against metabolically resistant *Ae. aegypti* mosquitoes. Moreover, metabolic assays should be conducted to better inform mosquito control programs for designing and implementing integrated vector management strategies.

## 1. Introduction

*Aedes aegypti* is the principal mosquito vector for many of the most medically significant arboviruses (arthropod-borne viruses) worldwide, including dengue, yellow fever, chikungunya, and Zika. Over the past couple of decades, the distribution of *Ae. aegypti* has increased globally [1]. This has led to concerns about potential corresponding increases in the distribution of *Aedes*-borne diseases. To mitigate the spread of *Aedes*-borne diseases, several mosquito control methods, such as elimination of potential immature mosquito habitats, biological control, and application of chemical insecticides, are employed independently or together within an integrated vector management strategy. However, the most extensively practiced control strategy for adult *Ae. aegypti*, especially during arbovirus outbreaks, is the application of chemical insecticides [2].

Pyrethroid-oriented vector control programs have increased worldwide to control adult *Ae. aegypti* and quell *Aedes*-borne disease epidemics [3]. Permethrin is the most widely used pyrethroid insecticide for controlling adult mosquitoes in the United States, which has led to a high frequency of resistance [4,5,6]. Insecticide resistance is a significant obstacle in the struggle against vector-borne diseases as it is a major contributing factor for the loss of efficacy of pyrethroids [7]. Resistance can promote operational failure of *Aedes*-borne disease control and subsequently lead to an increase in disease transmission [8,9]. Insecticide resistance in mosquitoes is often mediated by two broad mechanisms: mutations in the insecticide target proteins and enhanced metabolic detoxification of insecticides [10].

Metabolic detoxification of insecticides (metabolic resistance) is the process in which enzyme-mediated reactions cause the degradation or sequestration of insecticides before they can exert toxic effects. Increased expression of metabolically significant genes is a common adaptive mechanism contributing to insecticide resistance in mosquitoes [10,11,12,13,14]. The three major families of metabolic detoxification enzymes are cytochrome P450 monooxygenases (P450s), esterases (ESTs), and glutathione S-transferases (GSTs). The upregulation of enzymes belonging to these three families has been reported in a handful of mosquito species: *Culex pipiens* and *Cx. quinquefasciatus* [13,15,16,17,18], *Anopheles gambiae* and *An. funestus* [19,20,21,22,23], and *Ae. aegypti* [12,14,24,25,26,27].

To help overcome metabolic resistance, synergists that inhibit metabolic enzymes can be added to formulated pyrethroid products. Currently, piperonyl butoxide (PBO; P450 inhibitor) is the only metabolic enzyme inhibitor found in formulated pyrethroid products available for public health use. P450s assist in metabolism of toxic chemicals in insects by binding molecular oxygen and receiving electrons from NADPH to introduce an oxygen molecule into the substrate [28]. Many P450s are frequently associated with insecticide resistance in *Ae. aegypti* as they are commonly detected to be overexpressed in resistant strains globally. Furthermore, a handful of P450s have been functionally implicated in pyrethroid metabolization [11,29,30,31]. However, ESTs and GSTs have also been implicated in pyrethroid resistance in *Ae. aegypti* populations globally [9,12,13,14,25,27,32,33]. For ESTs, it is a series of reactions causing esterase hydrolysis of pyrethroids that results in insecticide detoxification [9]. Only one EST, a carboxy/cholinesterase, has been found capable of metabolizing pyrethroids [34]. On the other hand, GSTs are multifunctional enzymes that are involved in the metabolism, detoxification, and excretion of many exogenous and endogenous compounds [10,35]. A handful of GSTs are commonly over-transcribed in pyrethroid-resistant *Ae. aegypti* populations, but none have been shown to directly metabolize pyrethroids in mosquitoes [25,32,36]. One study showed that the use of diethyl maleate (DM; GST inhibitor) greatly increased the mortality rates of a permethrin-resistant *Ae. aegypti* population in Portugal [37]. However, neither DM nor S.S.S-tributyl phosphorotrithioate (DEF; EST inhibitor) are found in formulated pyrethroid products.

Because adulticides continue to be an imperative part of mosquito control, especially during arbovirus outbreaks, and permethrin is the pyrethroid adulticide active ingredient utilized most by control programs in our state, our first objective was to better understand statewide permethrin resistance in Florida *Ae. aegypti* populations. Additionally, we investigated the contribution of metabolic resistance by evaluating the effects of three metabolic enzyme inhibitors on the mortality of permethrin-resistant *Ae. aegypti* populations upon exposure to permethrin. This is the first known attempt of a statewide survey of metabolic resistance in Florida *Ae. aegypti*.

## 2. Materials and Methods

### 2.1. Mosquito Egg Collection

From 2019–2021, any interested Florida mosquito control program was provided an *Aedes* container-inhabiting mosquito egg surveillance kit. The contents of the *Aedes* container-inhabiting mosquito egg surveillance kit have been described previously in detail [38]. Briefly, they included surveillance instructions, 480-mL black plastic oviposition cups, seed germination paper, binder clips, and a microcentrifuge tube containing a 1:1 mixture of lactalbumin–yeast to be used as an oviposition attractant. Participating programs collected the seed germination papers from the field and sent them to the University of Florida, Institute of Food and Agriculture Sciences, Florida Medical Entomology Laboratory (UF/IFAS FMEL) (Table 1). Upon arrival, seed germination papers were allowed to airdry overnight if necessary. After drying, the number of viable eggs (not desiccated) on each paper was counted and recorded for each site. Egg papers from each site were stored in separate plastic containers with a damp cotton ball to prevent desiccation inside an insectary with conditions maintained at 27 °C ± 2 °C and 70% ± 5% relative humidity.

### 2.2. Mosquito Rearing

When sufficient numbers of viable F_0_ field *Aedes* eggs were collected (300 or more), egg papers were placed in 40.6 × 15.4 × 6.4 cm enamel pans containing approximately 2 L of tap water at a density of 250 viable eggs per rearing tray. A larval diet of lactalbumin–yeast was added ad libitum. Pupae originating from field-collected eggs were transferred from larval rearing trays to water-filled cups in 30.5 × 30.5 × 30.5 cm Bug Dorm adult rearing cages (Bioquip^®^, Rancho Dominquez, CA, USA). A cotton ball soaked with 10% sucrose solution was placed inside each cage as a carbohydrate source for adults that emerged. Adults were then sight-identified to species. Adult *Ae. albopictus* were removed from the cages using a mouth aspirator and discarded, leaving only *Ae. aegypti* adults.

To obtain enough mosquitoes from each population for testing, F_0_ adults of at least 3 days old were provided a bloodmeal twice per week from a live chicken (UF IACUC Protocol # 202007682) and were allowed to feed for 45 min. Immediately after blood feeding, a container with moist seed germination paper was placed inside the cage for the collection of F_1_ eggs. The germination paper was replaced every 7 days. If necessary, this process was repeated for F_1_ adults for the collection of the required number of F_2_ eggs. At no point were populations continued beyond the F_2_ generation.

All life stages of the mosquitoes were kept in a walk-in insectary at 27 °C ± 2 °C and 70% ± 5% relative humidity with a 12:12 LD photoperiod including a 1-h dusk and 1-h dawn phase. The “Orlando” (ORL1952) *Ae. aegypti* strain was reared under the same conditions described above for the field *Ae. aegypti* populations. This strain served as the susceptible reference as it has been a laboratory colony since 1952 [39].

### 2.3. Permethrin Susceptibility Assay

The Centers for Disease Control and Prevention (CDC) bottle bioassay was used to detect permethrin susceptibility or resistance in Florida *Ae. aegypti* populations. The CDC bottle bioassay was originally described by Brogdon and McAllister (1998), and we followed the protocol provided by the CDC [40,41]. Technical grade permethrin (100%, Chem Service Inc., West Chester, PA, USA) and acetone (Fisher Scientific, Waltham, MA, USA) were used to make 43 μg/mL permethrin stock solutions for the bottle bioassays, which is the permethrin diagnostic dose suggested by the CDC (Table 2). As part of each bioassay performed, 4 replicates of approximately 25 non-blood-fed 3-to-7-day old mosquitoes were exposed to 250-mL Wheaton^®^ bottles (Fisher Scientific) treated with 1 mL of the diagnostic dose of permethrin. Each bioassay also included a negative control, in which approximately 25 non-blood-fed 3-to-7-day old mosquitoes were exposed to a bottle treated only with a 1-mL acetone. The diagnostic time (DT; the time point at which 100% mortality was achieved for the susceptible population) was determined by conducting the CDC bottle bioassay with the susceptible ORL1952 *Ae. aegypti* reference strain. The diagnostic time was re-evaluated with every new stock of permethrin used to perform the CDC bottle bioassays. Permethrin stocks were remixed every 3 to 4 months if necessary [41].

Upon exposure to the bottles, mosquito mortality was recorded every 5 min for 15 min and then recorded every 15 min for a full 2 h. At the end of the 2-h bioassay, surviving mosquitoes were killed by freezing, and mosquitoes in bottles were counted in order to calculate the percent mortality at all time points. If the percent mortality in the negative control bottle was between 3 to 10%, mortality in treated bottles was corrected using Abbott’s formula [41,42]. If percent mortality in the control bottle was greater than 10%, the assay results were discarded. The susceptibility or resistance status of each *Ae. aegypti* population was determined by the population’s percent mortality at the DT determined using the susceptible ORL1952 reference strain and was classified according to CDC definitions. The *Ae. aegypti* populations were considered susceptible, developing resistance, or resistant if they experienced ≥ 97%, 90–97%, or <90% mortality, respectively, at the DT [41].

### 2.4. Permethrin Metabolic Resistance Assay

After a population was found to be permethrin-resistant, we used a variation of the CDC bottle bioassay to test for the presence of metabolic resistance by performing three additional permethrin bottle bioassays after exposing the mosquitoes to one of three metabolic enzyme inhibitors: piperonyl butoxide (PBO, which inhibits P450 activity); S.S.S-tributyl phosphorotrithioate (DEF, which inhibits EST activity); and diethyl maleate (DM, which inhibits GST activity), which were obtained from the CDC [41]. Briefly, we exposed approximately 125 mosquitoes to a bottle treated with 1 mL of the CDC’s suggested diagnostic dose of each inhibitor (Table 2) for 1 h. The mosquitoes were then transferred to holding cups and left to recover for 1 h. Following the 1-h recovery period, the mosquitoes were aspirated into the 4 respective permethrin-treated bottles and the 1 acetone-coated negative control bottle as we proceeded with the standard CDC bottle bioassay. Mortality was recorded every 5 min for 15 min and then every 15 min until 2 h. At the end of the 2-h assay, the mosquitoes were killed by freezing, then counted, and the percent mortality at all time points was calculated.

After testing with inhibitors, one of three outcomes could occur. In the first scenario, the population could return to full (or nearly full) susceptibility to permethrin. This indicates that metabolic resistance attributed by the class of enzymes acted on by the inhibitor was responsible for the resistance in that population. The second scenario is that the resistance to permethrin would only be partially abolished. This would suggest that the metabolic mechanisms related to the inhibitor are only partially conferring resistance and that other mechanisms may also play a role in resistance. Lastly, the third possible outcome is the resistance to permethrin would not change with pre-exposure to the inhibitor. This would imply that the mechanisms of resistance for this population are not appertaining to metabolic enzyme activity [41].

### 2.5. Statistical Analysis

All statistical analyses were performed using R version 4.0.4 (RStudio Inc., Boston, MA, USA). To determine the effects of the inhibitors PBO, DEM, and DEF on permethrin resistance for each *Ae. aegypti* population, we used the χ^2^ goodness of fit test to compare the number of mosquitoes alive or dead at 30 min for bottle bioassays performed with permethrin, PBO + permethrin, DEM + permethrin, and DEF + permethrin [6,43,44,45,46]. The significance level was set at 5%. Significant effects were further analyzed by all possible pairwise comparisons of distributions using χ^2^ tests (α value adjusted for multiple comparisons using the sequential Bonferroni method [47]).

## 3. Results

### 3.1. Permethrin Susceptiblity

**Figure 1:**
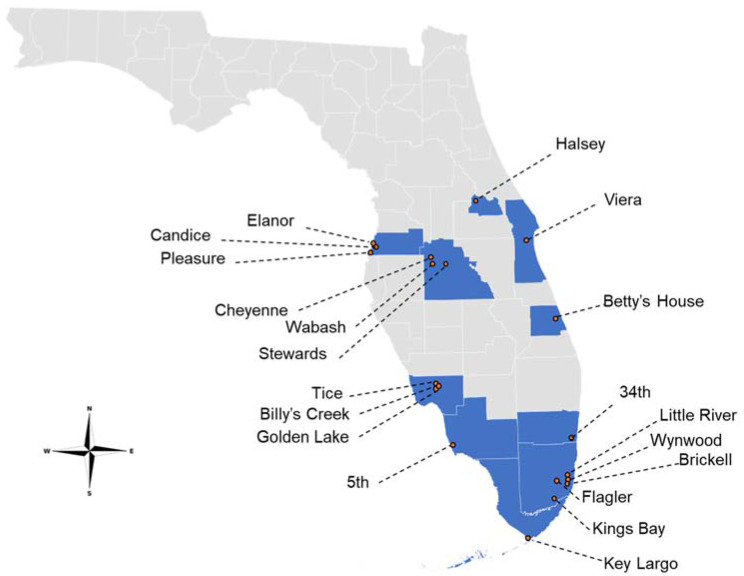
Collection locations of Florida *Aedes aegypti* used in this study (2019–2020).

A total of 20 *Ae. aegypti* populations from 10 Florida counties were assayed for permethrin susceptibility/resistance (Figure 1). The percent mortality at the DT for all populations was less than 90%; therefore, they were classified as resistant (R) (Table 3) [41]. The average percent mortality at the DT for all populations was 11.6%. The Candice population exhibited the highest percent mortality (54.5%), and there were four populations that exhibited the lowest percent mortality at the DT (0%): 5th, Flagler, Kings Bay, and Cheyenne (Table 3).

### 3.2. Permethrin Metabolic Resistance

When compared to the results of the permethrin-only bottle bioassays, 11 of the 20 (55%) *Ae. aegypti* populations exhibited significant increases (All *p* < 0.025) in percent mortality at 30 min when pre-exposed to at least one inhibitor (Table 4). Specifically, 8 of 11 (73%) populations had significant increases in percent mortality at 30 min attributed to DEF exposure, 7 of the 11 (64%) to PBO, and 6 of 11 (55%) to DM. Not only were there significant differences in percent mortality when comparing permethrin-only treatments to treatments with pre-exposure to the inhibitors, but there were also significant differences in inhibitor performance compared to one another. For example, in the Viera and Wynwood *Ae. aegypti* populations, all three inhibitors significantly increased percent mortality (*p* < 0.013) with PBO, DEF, and DM contributing equally with no significant increase in mortality caused by one inhibitor compared to the other. However, for the Brickell population, PBO yielded a significant increase in mortality (*p* < 0.01) compared to DM. For three populations (5th, Cheyenne, and Wabash), there was a significant increase in the mortality (*p* < 0.025) caused by DEF as compared to either PBO or DM. Both DEF and DM resulted in significantly higher mortality rates compared to PBO for the Billy’s Creek (*p* < 0.017) and Key Largo populations (*p* < 0.013). Additionally, for the Key Largo population, DEF contributed to a significantly higher mortality than DM (*p* < 0.017) (Table 4).

## 4. Discussion

Pyrethroids are among the most widely used vector control tools worldwide and are the most extensively applied class of insecticides for control of adult *Ae. aegypti* [48,49]. Unfortunately, due to years of overuse, resistance to pyrethroids in *Ae. aegypti* has become a global issue. Resistance to pyrethroids is a threat to the efficacy of adulticiding, which can promote operational failure of *Aedes*-borne disease control and subsequently lead to an increase in disease transmission. In the United States, permethrin, a type I pyrethroid, is the most common insecticide used to control *Ae. aegypti* [5]. To assess the frequency of resistance in local Florida *Ae. aegypti* populations, we performed susceptibility assays with permethrin on 20 populations throughout the state. Similar to other permethrin susceptibility studies performed with *Ae. aegypti* in Florida, all populations tested were found to be resistant [4,6].

To better understand the mechanisms responsible for permethrin resistance in Florida *Ae. aegypti*, we evaluated the impact of the metabolic enzyme inhibitors PBO (inhibits P450 activity), DM (inhibits GST activity), and DEF (inhibits EST activity) on permethrin resistance in each population. We found that pre-exposure to PBO, DEF, and/or DM led to significant increases in the mortality against permethrin for multiple Florida *Ae. aegypti* populations, partially returning them to a more susceptible status.

Currently, PBO is the only metabolic enzyme inhibitor that is found in formulated pyrethroid products available for public health use. Other studies performed on pyrethroid resistant *Ae. aegypti* and *An. gambiae* found that pre-exposure to PBO lead to a significant enhancement of susceptibility to pyrethroids (permethrin and/or deltamethrin) [43,44,45,46,50,51]. This is similar to what we found in Florida *Ae. aegypti* populations against permethrin. Dadzie et al. (2017) also noted that, with highly susceptible *An. gambiae* populations, PBO had no effect. Although all of the populations tested in this study were classified as resistant under the CDC susceptibility guidelines, we saw a similar trend where populations with higher permethrin percent mortality values at 30 min (>81%) often had no significant increase in mortality when pre-exposed to PBO (i.e., 34th, Golden Lake, Flagler, Little River, Key Largo, Candice, Stewards) [44]. In contrast, Dadzie et al. (2017) also found that, for the opposite extreme (highly resistant populations), the ability of PBO to synergize pyrethroid insecticides seemed to diminish [44]. This was not observed in Florida *Ae. aegypti* populations, as the two most resistant populations, Viera and Cheyenne, each demonstrated <30% mortality after 30 min of exposure to permethrin and still demonstrated a substantially significant increase in mortality attributed to PBO (*p* = 0.013).

Furthermore, we found that DEF pre-exposure increased susceptibility to permethrin in a greater number of *Ae. aegypti* populations compared to PBO pre-exposure. This is an interesting discovery considering that the oxidative stress propagated by P450s is thought to be the most important contributor to metabolic resistance in insects [27,30,33,35,52]. Typically, esterase activity is known for conferring organophosphate and carbamate resistance in mosquitoes [53,54], although some studies revealed that pyrethroid resistance was the outcome of elevated EST activity in *Ae. aegypti* [55,56]. Additionally, Lucas et al. (2020) identified pyrethroid resistance attributed to both oxidase and esterase activity in Collier County, FL *Cx. quinquefasciatus* mosquitoes. Results from the latter studies are comparable to what we found in this study where inhibiting esterase activity via DEF inhibitor contributed to partially overcoming pyrethroid resistance [57]. Often, studies investigating the effects of metabolic enzyme inhibition on pyrethroid-resistant strains of mosquitoes found that PBO had a larger impact on mortality than DEF [40,43,58]. However, Xu et al. (2005) performed a study with *Cx. quinquefasciatus* and found that DEF had a greater impact on permethrin-resistant populations than PBO and DM [59]. Nonetheless, all three enzyme inhibitors significantly increased susceptibility to the pyrethroids (permethrin and/or deltamethrin) [59]. Interestingly, PBO has also been found to inhibit resistance-associated esterases in many insects [60,61,62]. While PBO has been shown to inhibit esterases in addition to oxidases in some insects, our study demonstrated pre-exposure to DEF led to a significant increase in a higher percentage (73%) of the permethrin resistant *Ae. aegypti* populations compared to PBO (64%). This suggests that PBO may not be inhibiting all esterases in *Ae. aegypti*, and formulated pyrethroid products containing PBO might benefit from the addition of another esterase inhibitor, such as DEF.

Although for not as many populations as PBO or DEF, we found that DM also contributed to increasing the susceptibly to lethality against permethrin in some of the *Ae. aegypti* populations tested. This suggests that GSTs may be contributing to metabolic resistance in these populations. Because DEF and DM are not added to formulated products for public health use, there are fewer studies testing their efficacy than with PBO. Similar to the results of this study, Seixas et al. (2017) and Rigby et al. (2020) found that pre-exposure to DM significantly increased mortality of permethrin-resistant *Ae. aegypti* to permethrin [37,46]. Though, in our study, pre-exposure to DM did not result in a significant increase in mortality compared to PBO and DEF for any of the *Ae. aegypti* populations tested. Instead, DM consistently had a lesser or parallel effect as PBO or DEF.

Not all the permethrin-resistant *Ae. aegypti* populations that we tested exhibited metabolic resistance. These findings suggest that other mechanisms (such as kdr) are responsible for conferring permethrin resistance in these populations. Of the populations that did exhibit significant increases in mortality following inhibitor pre-exposure, not all inhibitors performed equally. Based on these results, we can infer that the contribution to metabolic resistance by these three families of metabolic enzymes differs among Florida populations of *Ae. aegypti* and mechanism testing should take place to provide useful information for insecticide-resistance management strategies. Information on population genetics (i.e., DNA sequencing) for these populations could provide improved insight for potential underlying genetics influencing the difference in outcomes between permethrin susceptibility and inhibitor effectiveness among these populations. Although further investigation is needed to better understand the implications for vector control strategies, our results suggest that other metabolic enzymes apart from P450s (i.e., ESTs and GSTs) could be involved in the development of resistance to pyrethroids. Thus, vector control products that incorporate DEF or DM into their pyrethroids, particularly permethrin products, may be useful in increasing the efficacy of permethrin against permethrin-resistant *Ae. aegypti*.

## 5. Conclusions

These results have shown that mosquito metabolic resistance testing using inhibitors should become a more common practice for mosquito control agencies. Additionally, if DEF and DM increase the efficacy of permethrin against resistant *Ae. aegypti* mosquitoes similarly to or better than PBO, as suggested by our results, their inclusion in future pyrethroid formulated products should be considered.

## Figures and Tables

**Table 1:** Collection information for Florida *Aedes aegypti* used in this study.

County	Site	Latitude	Longitude	Date Collected	Collector
Brevard	Viera	28.24641	−80.73662	10–11/2019	C. McDowell
Broward	34th	26.035359	−80.178085	10/2020	T. Hamilton
Collier	5th	26.14090	−81.803287	10/2020	R. Bales
Lee	Billy’s Creek	26.660952	−81.811921	08/2020	K. Tyler-Julian
Lee	Golden Lake	26.65284	−81.81183	08/2020	K. Tyler-Julian
Lee	Tice	26.67116	−81.811934	10/2020	K. Tyler-Julian
Miami Dade	Brickell	25.7586	−80.19819	06–07/2020	J. Medina
Miami Dade	Flagler	25.77342	−80.28567	09–11/2019	J. Medina
Miami Dade	Kings Bay	25.63456	−80.29749	09–11/2019	J. Medina
Miami Dade	Little River	25.83762	−80.19921	07–08/2019	J. Medina
Miami Dade	Wynwood	25.80475	−80.19776	07–08/2019	J. Medina
Monroe	Key Largo	25.131444	−80.405158	06/2020	C. Pruszynski
Pasco	Candice	28.261939	−82.705499	09–10/2020	A. Janusauskaite
Pasco	Elanor	28.322423	−82.706873	09–10/2020	A. Janusauskaite
Pasco	Pleasure	28.186338	−82.745269	09–10/2020	A. Janusauskaite
Polk	Cheyenne	28.14449	−82.00326	08/2020	J. Mosley
Polk	Stewards	28.04472	−81.77675	08/2020	J. Mosley
Polk	Wabash	28.04866	−81.99078	08/2020	J. Mosley
Seminole	Halsey	28.82609	−81.335136	10/19–01/20	T. Jones
St. Lucie	Betty’s House	27.411226	−80.336365	08/2020	B. Starke

**Table 2:** Dose of chemicals used for CDC bottle bioassays as per CDC guidelines.

Chemical	Diagnostic Dose (μg/mL)
Permethrin	43
Diethyl maleate (DM)	80
Piperonyl butoxide (PBO)	400
S.S.S-tributyl phosphorotrithioate (DEF)	125

**Table 3:** Resistance status of *Aedes aegypti* populations determined by % mortality at the diagnostic time (DT).

County	Site	DT (min)	% Mortality at DT	Resistance Status	Generation Tested
Brevard	Viera	10	6	R	F1
Broward	34th	10	21	R	F0
Collier	5th	10	0	R	F1
Lee	Billy’s Creek	10	1	R	F1
Lee	Golden Lake	10	38	R	F1
Lee	Tice	10	7	R	F1
Miami Dade	Brickell	10	1	R	F2
Miami Dade	Flagler	10	0	R	F1
Miami Dade	Kings Bay	10	0	R	F2
Miami Dade	Little River	15	11	R	F1
Miami Dade	Wynwood	10	5	R	F1
Monroe	Key Largo	10	15	R	F2
Pasco	Candice	10	55	R	F2
Pasco	Elanor	10	13	R	F1
Pasco	Pleasure	10	4	R	F1
Polk	Cheyenne	10	0	R	F1
Polk	Stewards	10	11	R	F2
Polk	Wabash	10	2	R	F2
Seminole	Halsey	10	42	R	F2
St. Lucie	Betty’s House	10	1	R	F1

DT = diagnostic time, R = resistant.

**Table 4:** Population-specific percent mortality at 30-min post-exposure to permethrin in Florida *Aedes aegypti* populations that were exposed to permethrin only or exposed to 1 of 3 metabolic enzyme class inhibitors, piperonyl butoxide (PBO), S.S.S-tributyl phosphorotrithioate (DEF), and diethyl maleate (DM), prior to permethrin exposure.

County	Site	Insecticide	No. Tested	% Mortality (30 min)	*X* ^2^	Critical *p* Value	*p* Value
Brevard	Viera	Permethrin	75	26	-	-	-
		PBO + Permethrin	83	74	37.8	0.013	**7.90 × 10^−10^**
		DEF + Permethrin	63	84	47.3	0.01	**6.15** ** × 10^−12^**
		DM + Permethrin	100	87	69.8	0.008	**<** **2.2 × 10^−16^**
Broward	34th	Permethrin	100	81	-	-	-
		PBO + Permethrin	115	77	0.42	0.025	5.17 × 10^−1^
		DEF + Permethrin	131	72	2.24	0.008	1.35 × 10^−1^
		DM + Permethrin	104	77	0.52	0.017	4.72 × 10^−1^
Collier	5th	Permethrin	87	38	-	-	-
		PBO + Permethrin	108	41	0.16	0.05	6.90 × 10^−1^
		DEF + Permethrin	104	63	12.37	0.01	**4.37** ** × 10^−4^**
		DM + Permethrin	107	26	3.08	0.025	7.93 × 10^−2^
Lee	Billy’s Creek	Permethrin	134	57	-	-	-
		PBO + Permethrin	134	71	5.83	0.025	**1.57** ** × 10^−2^**
		DEF + Permethrin	117	92	40.44	0.008	**2.03** ** × 10^−10^**
		DM + Permethrin	143	87	30.65	0.01	**3.09** ** × 10^−8^**
	Golden Lake	Permethrin	117	91	-	-	-
		PBO + Permethrin	99	85	2.29	0.025	1.31 × 10^−1^
		DEF + Permethrin	127	98	4.62	0.01	3.16 × 10^−2^
		DM + Permethrin	119	92	0.08	0.05	7.81 × 10^−1^
	Tice	Permethrin	143	57	-	-	-
		PBO + Permethrin	120	33	15.33	0.01	**9.03** ** × 10^−5^**
		DEF + Permethrin	104	43	4.31	0.017	3.79 × 10^−2^
		DM + Permethrin	112	27	22.78	0.008	**1.82** ** × 10^−6^**
Miami-Dade	Brickell	Permethrin	132	58	-	-	-
		PBO + Permethrin	102	77	10.16	0.008	**1.43** ** × 10^−3^**
		DEF + Permethrin	84	62	0.4	0.017	5.28 × 10^−1^
		DM + Permethrin	99	59	0.02	0.05	8.78 × 10^−1^
	Flagler	Permethrin	72	82	-	-	-
		PBO + Permethrin	96	83	0.06	0.05	8.14 × 10^−1^
		DEF +Permethrin	93	91	3.27	0.017	7.08 × 10^−2^
		DM + Permethrin	88	58	10.61	0.013	**1.13** ** × 10^−3^**
	Kings Bay	Permethrin	120	50	-	-	-
		PBO + Permethrin	97	57	0.97	0.008	3.25 × 10^−1^
		DEF + Permethrin	81	49	0.01	0.05	9.32 × 10^−1^
		DM + Permethrin	114	45	0.65	0.013	4.20 × 10^−1^
	Little River	Permethrin	85	98	-	-	-
		PBO + Permethrin	97	89	5.52	0.01	1.88 × 10^−2^
		DEF + Permethrin	93	85	8.76	0.008	**3.08** ** × 10^−3^**
		DM + Permethrin	92	91	3.33	0.013	6.79 × 10^−2^
	Wynwood	Permethrin	131	58	-	-	-
		PBO + Permethrin	156	84	23.87	0.01	**1.03** ** × 10^−6^**
		DEF + Permethrin	127	88	26.7	0.008	**5.05** ** × 10^−8^**
		DM + Permethrin	142	83	23.04	0.013	**1.59** ** × 10^−6^**
Monroe	Key Largo	Permethrin	135	84	-	-	-
		PBO + Permethrin	99	74	3.57	0.05	5.90 × 10^−2^
		DEF + Permethrin	124	100	21.75	0.01	**3.41** ** × 10^−6^**
		DM + Permethrin	122	94	6.7	0.025	**9.76** ** × 10^−3^**
Pasco	Elanor	Permethrin	102	70	-	-	-
		PBO + Permethrin	105	88	10.03	0.008	**1.54** ** × 10^−3^**
		DEF + Permethrin	100	86	7.84	0.01	**5.12** ** × 10^−3^**
		DM + Permethrin	75	80	2.43	0.013	1.19 × 10^−1^
	Pleasure	Permethrin	122	59	-	-	-
		PBO + Permethrin	118	63	0.34	0.025	5.58 × 10^−1^
		DEF + Permethrin	111	73	5.02	0.01	2.50 × 10^−2^
		DM + Permethrin	112	55	0.32	0.05	5.72 × 10^−1^
	Candice	Permethrin	46	91	-	-	-
		PBO + Permethrin	122	98	4.83	0.017	2.80 × 10^−2^
		DEF + Permethrin	-	-	-	-	-
		DM + Permethrin	93	98	3.19	0.025	7.40 × 10^−2^
Polk	Cheyenne	Permethrin	95	14	-	-	-
		PBO + Permethrin	125	70	68.07	0.013	**<** **2.2 × 10^−16^**
		DEF + Permethrin	139	88	129.73	0.008	**<** **2.2 × 10^−16^**
		DM + Permethrin	128	78	90.59	0.01	**<2.2** ** × 10^−16^**
	Stewards	Permethrin	122	85	-	-	-
		PBO + Permethrin	124	93	3.54	0.025	6.00 × 10^−2^
		DEF + Permethrin	52	69	5.95	0.01	1.47 × 10^−2^
		DM + Permethrin	106	85	0.01	0.05	9.43 × 10^−1^
	Wabash	Permethrin	134	49	-	-	-
		PBO + Permethrin	120	41	1.81	0.05	1.78 × 10^−1^
		DEF + Permethrin	115	72	13.53	0.013	**2.35** ** × 10^−4^**
		DM + Permethrin	145	32	8.2	0.017	**4.20** ** × 10^−3^**
Seminole	Halsey	Permethrin	127	82	-	-	-
		PBO + Permethrin	118	93	7.11	0.008	**7.69** ** × 10^−3^**
		DEF + Permethrin	95	83	0.06	0.05	8.06 × 10^−1^
		DM + Permethrin	79	85	0.29	0.017	5.87 × 10^−1^
St. Lucie	Betty’s House	Permethrin	117	40	-	-	-
		PBO + Permethrin	110	55	5.3	0.01	2.12 × 10^−2^
		DEF + Permethrin	127	52	3.41	0.013	6.49 × 10^−2^
		DM + Permethrin	118	64	7.87	0.008	**5.02** ** × 10^−3^**

**Bolded***p*-values are significant (≤ critical *p* value).

## Data Availability

Data sharing is not applicable to this article.
